# *Rickettsia massiliae* in the Canary Islands

**DOI:** 10.3201/eid1511.090681

**Published:** 2009-11

**Authors:** Isabel G. Fernández de Mera, Zorica Zivkovic, Margarita Bolaños, Cristina Carranza, José Luis Pérez-Arellano, Carlos Gutiérrez, José de la Fuente

**Affiliations:** Instituto de Investigación en Recursos Cinegéticos IREC (Consejo Superior de Investigaciones Cientificas–Universidad de Castilla-La Mancha–Junta de Comunidades de Castilla-La Mancha), Ciudad Real, Spain (I.G. Fernández de Mera, J. de la Fuente); Utrecht University, the Netherlands (Z. Zivkovic); Hospital Universitario Insular de Gran Canaria, Canary Islands, Spain (M. Bolaños, C. Carranza, J.L. Pérez-Arellano); Universidad de Las Palmas de Gran Canaria, Canary Islands (J.L. Pérez-Arellano, Carlos Gutiérez); Oklahoma State University, Stillwater, Oklahoma, USA (J. de la Fuente).

**Keywords:** Rickettsia massiliae, rickettsia, Canary Islands, ticks, letter

**To the Editor**: *Rickettsia massiliae* was recently recognized as a human tick-borne spotted fever group rickettsia (*1*). We report the finding of *R. massiliae* in *Rhipicephalus pusillus* ticks from Gran Canaria, Canary Islands, Spain. Introduction of this pathogen into the Canary Islands is thought to have resulted from translocation of the European wild rabbit *Oryctolagus cuniculus* (Linnaeus), a preferred host of *R. pusillus* ticks (www.kolonin.org/16_4.html), from the Iberian Peninsula 600 years ago (*2*).

We collected questing adult ticks in 2008 in Gran Canaria and identified 2 tick species, *Hyalomma lusitanicum* (n = 82 [46 females]) and *R. pusillus* (n = 8 [5 females]). Whole ticks were preserved in 70% ethanol and used for DNA extraction by using TriReagent (Sigma, St. Louis, MO, USA) according to the manufacturer’s instructions. We identified rickettsial sequences by using PCR primers that amplify fragments of 16S rRNA, *ompB, atpA, dnaA, dnaK*, and *recA* genes (Table). Amplicons were cloned into pGEM-T (Promega, Madison, WI, USA), and 3 independent clones were sequenced from both ends for each gene marker. Sequence similarity search was performed by using BLAST (www.ncbi.nlm.nih.gov). Rickettsial DNA was detected in 2 *R. pusillus* males only; sequences were identical in both ticks. Fragments of 16S rRNA were 99% identical to the *R. massiliae* strain Mtu5 (CP000683) isolated from *R. sanguineus* ticks in southern France (*3*), and fragments of *ompB, atpA, dnaA, dnaK*, and *recA* genes were 100% identical to the *R. massiliae* strain Bar29 (AF123710, AY124739, DQ821798, DQ821828, and AY124750, respectively), previously isolated from *R. sanguineus* ticks in Catalonia, Spain (*4*) (Table).

**Table:** *Rickettsia massiliae* PCR conditions and amplicon size, Canary Islands, 2008*

Gene	Description (GenBank accession no.)	Primer sequence (5′ → 3′)	Amplicon size, bp	PCR annealing conditions
16S rRNA	16S ribosomal RNA (GQ144453)	F: AGAGTTTGATCCTGGCTCAG R: AACGTCATTATCTTCCTTGC	416	50°C/30 s
*ompB*	Outer membrane protein (GQ144450)	F: GGGTGCTGCTACACAGCAGAA R: CCGTCACCGATATTAATTGCC	618	53°C/30 s
*dnaK*	Heat-shock protein 70 (GQ144451)	F: AGCGTCAAGCAACGAAAGAT R: CAAACGTTGAAGTGCTAAAGG	323	50°C/30 s
*dnaA*	Chromosomal replication initiation protein (GQ144449)	F: CCTACTAACTTTGTTAGAGATT R: TGATGATTCTGCAACCGCTC	241	56°C/30 s
*recA*	RecA recombination protein (GQ144452)	F: TGCTTTTATTGATGCCGAGC R: CTTTAATGGAGCCGATTCTTC	428	52°C/30 s
*atpA*	ATP synthase F1 alpha subunit (GQ144448)	F: ACATATCGAGATGAAGGCTCC R: CCGAAATACCGACATTAACG	731	48°C/30 s

*GenBank accession numbers correspond to *R. massiliae* sequences identified in this study. PCRs were completed by employing the Access RT-PCR system (Promega, Madison, WI, USA) with 1 ng DNA, the oligonucleotide primers, and annealing conditions and with extension for 1 min at 68ºC. F, forward; R, reverse.

*R. massiliae* was first isolated in 1992 from *R. sanguineus* ticks collected near Marseille, France (*5*). Since then, the pathogen has been identified in different *Rhipicephalus* species in France, Greece, Portugal, Switzerland, Spain, North and Central Africa, Argentina, and the United States (*6**,**7*). *R. massiliae* has been identified in southern Spain (*8*) but not in the Canary Islands. *R. pusillus* ticks are commonly found in southern Europe (Portugal, Spain, and France) and northern Africa (Tunisia and Morocco). All stages of these ticks inhabit burrows of wild rabbits and feed on them (www.kolonin.org/16_4.html).

Wild rabbits were introduced into the Canary Islands at the end of 14th century during colonization by the kingdom of Castilla. Colonists were asked to bring rabbit couples with them to provide food in the islands (*2*), a practice continued by new colonists because of their interest in hunting this rabbit species. Introduction of wild rabbits by colonists led to establishment of parasites, such as helminths, coccidia, and viruses in the Canary Islands (*9*). *R. pusillus*, a common ectoparasite (tick) that feeds on wild rabbits on the Iberian Peninsula, was also introduced this way. *R. massiliae* could have been introduced in the islands by infected *R. pusillus* ticks or by infected wild rabbits if this species serves as a natural reservoir host for the pathogen.

To find evidence for this hypothesis, we tested blood and liver samples of 150 wild rabbits from both Canary Islands and Andalucía (southern Spain) by using *Rickettsia*-specific PCR primers (Table). No *R. massiliae* DNA was detected in the rabbit samples tested, suggesting that the pathogen probably was introduced in the Canary Islands with infected *R. pusillus* ticks feeding on rabbits. Alternatively, *R. massiliae* infection levels in wild rabbits may be below the PCR detection limit and were not detected.

The Canary Islands are a popular tourist destination. The presence of *R. massiliae* in the islands constitutes a risk for human infection and should be considered in hospital diagnostic and wildlife management strategies. As with other *Rhipicephalus* spp., *R. pusillus* ticks could feed on humans under certain circumstances (*10*). Our results emphasize the risks associated with unsupervised animal translocations, a factor that probably plays a role in the introduction of ticks and tick-borne pathogens in different parts of the world.
